# Factors associated with sub-microscopic placental malaria and its association with adverse pregnancy outcomes among HIV-negative women in Dar es Salaam, Tanzania: a cohort study

**DOI:** 10.1186/s12879-020-05521-6

**Published:** 2020-10-27

**Authors:** Aneth Vedastus Kalinjuma, Anne Marie Darling, Ferdinand M. Mugusi, Ajibola Ibraheem Abioye, Fredros O. Okumu, Said Aboud, Honorati Masanja, Davidson H. Hamer, Ellen Hertzmark, Wafaie W. Fawzi

**Affiliations:** 1 Department of Intervention and Clinical Trials, Ifakara Health Institute, P.O. Box 53, Ifakara, Morogoro, Tanzania; 2 Department of Global Health and Population, Harvard T.H. Chan School of Public Health, Boston, MA USA; 3 Departments of Internal Medicine; and Microbiology and Immunology, School of Medicine, Muhimbili University of Health and Allied Sciences, Dar-es-Salaam, Tanzania; 4 Department of Global Health, Boston University School of Public Health, Boston, MA USA; 5 Section of Infectious Diseases, Department of Medicine, Boston University School of Medicine, Boston, MA USA

**Keywords:** Sub-microscopic parasitemia, placental malaria, low birth weight, Premature, Small for gestational age, Histopathology, DNA PCR

## Abstract

**Background:**

Malaria infection during pregnancy has negative health consequences for both mothers and offspring. Sub-microscopic malaria infection during pregnancy is common in most African countries. We sought to identify factors associated with sub-microscopic placental malaria, and its association with adverse pregnancy outcomes among HIV-negative pregnant women in Dar es Salaam, Tanzania.

**Methods:**

We recruited a cohort of pregnant women during their first trimester and assessed for the occurrence of placental malaria and pregnancy outcomes. The follow-up was done monthly from recruitment until delivery. Histopathology placental malaria positive results were defined as the presence of malaria pigment or parasitized erythrocytes on the slide (histology-positive (HP)), and the sub-microscopic placental infection was defined as positive *Plasmodium falciparum* DNA by polymerase chain reaction (DNA PCR) amplification in a negative histopathology test. Adverse pregnancy outcomes investigated included low birth weight (birth weight below 2.5 kg), prematurity (live birth below 37 weeks), and small-for-gestational-age (SGA) (live born with a birth weight below 10th percentile for gestational age and sex). Weighted baseline category logit, log-binomial, and log-Poisson models were used to assess factors associated with placental malaria, and its association with adverse pregnancy outcomes.

**Results:**

Among 1115 women who had histopathology and DNA PCR performed, 93 (8%) had HP placental infection, and 136 (12%) had the sub-microscopic placental infection. The risk of sub-microscopic placental malaria was greater in women who did not use mosquito prevention methods such as bed nets, fumigation, or mosquito coils (odds ratio (OR) = 1.75; 95% confidence interval (CI): 1.05–2.92; *P* = 0.03) and in women who were anemic (OR = 1.59; 95% CI: 1.20–2.11; *P* = 0.001). Women who were underweight had reduced odds of sub-microscopic placental malaria infection (OR = 0.33; 95% CI: 0.17–0.62; P = 0.001). Women who were overweight/obese had 1.48 times higher the odds of HP placental malaria compared to normal weight (OR = 1.48; 95% CI: 1.03–2.11; *P* = 0.03). HP placental malaria infection was associated with an increased risk of SGA births (RR = 1.30, 95% CI: 0.98–1.72, *P* = 0.07). In contrast, the sub-microscopic infection was associated with a reduced risk of SGA births (RR = 0.61, 95% CI: 0.43–0.88, *P* = 0.01). Placental malaria was not associated with low birth weight or prematurity.

**Conclusion:**

Malaria prevention methods and maternal nutrition status during early pregnancy were important predictors of sub-microscopic placental malaria. More research is needed to understand sub-microscopic placental malaria and the possible mechanisms mediating the association between placental malaria and SGA.

## Background

Worldwide, 75,000 to 200,000 infant deaths every year are attributable to malaria infection during pregnancy (MiP) [1]. Approximately 25 million pregnant women are at risk of *Plasmodium falciparum (P. falciparum)* infections each year in sub-Saharan Africa [2]. Twenty-six percent of severe anemia during pregnancy is attributable to MiP, and the proportion of maternal deaths linked to malaria infections ranges from 0.5 to 23% in hospital-based studies and 3 to 18% in community-based studies [1, 2]. MiP also increases the risk of various adverse pregnancy outcomes such as low birth weight (LBW), prematurity, intrauterine growth retardation (IUGR), and stillbirth [1–3]. Further, the offspring of mothers diagnosed with placental malaria also have an increased risk of all-cause anemia and malaria infection during infancy [4, 5].

Histopathology examination of stained placental biopsies is considered as the gold standard for diagnosis of placental malaria (hereafter referred to as histology-positive (HP) placental malaria), but this test is impractical or not available in some settings [6, 7]. Previous studies have pointed out that pregnant women often have low parasite densities, and the use of highly sensitive diagnostic methods such a DNA polymerase chain reaction (DNA PCR) (referred to as sub-microscopic placental malaria) has been recommended to enhance detection of placental malaria [6]. Sub-microscopic MiP remains common in most African countries including Tanzania [8–11], even though there was a 41% reduction of the malaria incidence rate in sub-Saharan Africa for the period of 2000–2015 [12]. The decreasing proportion of individuals presenting with clinical malaria infection over time has been linked with the scale-up of effective malaria interventions including insecticide-treated nets, effective case management, and indoor residual spraying [13].

Peripheral sub-microscopic malaria is strongly associated with placental malaria and low maternal hemoglobin levels [14, 15], but there are contradictory findings about the association between placental malaria and adverse pregnancy outcomes [11, 16, 17]. Cottrell et al. [16] found that sub-microscopic malaria infection during early pregnancy was associated with adverse pregnancy outcomes, whereas infection detected at delivery in the placenta and peripheral blood sample was not. In contrast, Cohee et al. [14] did not find an association between adverse pregnancy outcomes and peripheral sub-microscopic infection detected during pregnancy, and at delivery, and sub-microscopic placental malaria. As a result, the WHO has recommended more studies to investigate the association between sub-microscopic infection and adverse pregnancy outcomes [18].

Limited evidence is available on the association between adverse pregnancy outcomes and placental malaria infections (particularly sub-microscopic variant) in tropical African cities such as Dar-es-Salaam, Tanzania, where the malaria burden has been significantly reduced, but low-level transmission remains. We, therefore designed a study with the objectives of identifying factors associated with sub-microscopic placental malaria and examined its association with LBW, prematurity, and small-for-gestational-age (SGA) births among HIV-negative pregnant women in their first and second pregnancy in Dar Es Salaam, Tanzania.

## Methods

### Study design and participants

We used data from a trial that examined the effect of prenatal vitamin A and zinc supplements on malaria in pregnancy among women in their first or second pregnancy [19]. In brief, women were randomized to placebo (625 women), vitamin A alone (625 women), zinc alone (625 women), and vitamin A and zinc (625 women). Women were enrolled in their first trimester (below 13 weeks of gestation) and followed up monthly until delivery for placental malaria and adverse pregnancy outcomes. Gestational age was estimated using the last menstrual period. Recruitment occurred between December 2010 and September 2013, and follow-up ended in June 2014. Trial recruitment sites were in Ilala and Temeke districts, Dar-es-Salaam, Tanzania. All women included in the trial received iron (60 mg daily) and folic acid (5 mg daily) supplements, a voucher for insecticide-treated bed nets, and sulphadoxine-pyrimethamine (SP) (1500 mg sulfadoxine and 75 mg pyrimethamine) as per Tanzanian standard of care.

Socio-demographic and clinical variables were assessed at recruitment (baseline); these include age, parity, twin pregnancy status, years of education, marital status, gestational age, employment status, and use of fumigation, mosquito coil or bed nets at night. Selected possessions were asked about, from which a Filmer-Pritchet wealth index was computed [20]. Body mass index (BMI) (weight in kg divided by height (in meters) squared) and hemoglobin (in g/dL) were assessed. Age was classified into ≤24, 25–34 and ≥ 35 years, and years of education were categorized into 0–7 (no education to primary school education), 8–11 (secondary school education), and ≥ 12 years (high school and above education). The wealth index was grouped into quartiles, BMI was classified as < 18.5 (underweight), 18.5–24.9 (normal weight) or ≥ 25 kg/m^2^ (overweight/obese) [21], and hemoglobin was classified as ≥11.0 g/dL (not anemic) or < 11.0 g/dL (anemic) [22].

### Outcomes assessed at delivery

Placental malaria was evaluated using both histopathology and DNA PCR. HP placental malaria was defined as the presence of malaria pigment or parasitized erythrocytes on the slide, and sub-microscopic placental malaria infection was defined as positive *P. falciparum* DNA by PCR amplification in samples negative for histopathology test. Details on the two methods have been previously described [19]. Briefly, tissue from fresh sampled placentas was collected during delivery and the collected sample was divided for both uses. Histopathologic infections were defined as the presence of malaria pigment or parasitized erythrocytes on tissue from a central parenchymal section of the placenta. Placenta samples were stained and then examined using polarized light microscopy. A single placental histopathologist classified all slides, and quality control of diagnoses were done externally in a subset of 100 slides.

For the nucleic acid studies, tissue was stabilized in RNA later (Qiagen, Hilden, Germany) and homogenized. Genomic DNA was extracted using DNeasy (Qiagen). Taqman® qRT-PCR (Thermo Fisher Scientific, Waltham, MA) was used for amplification using published primer and probe sets (*P. falciparum*-specific [23] and general *Plasmodium* 18S rRNA genes [24]). The tissue was then tested for PCR inhibitors. Positive and negative controls were included on each plate for quality, and the minimum parasite density detection level was 0.000362 parasites/μL [24]. At recruitment and during follow-up peripheral malaria infection was diagnosed using microscopy of thin and thick Giemsa staining blood smears.

Adverse pregnancy outcomes investigated in this study were LBW (birth weight below 2.5 kg), SGA births (live birth weight below 10^th^ percentile for the exact day of gestation and sex based on INTERGROWTH-21^st^ standards [25, 26]) as a proxy outcome of IUGR, and prematurity (live birth before 37 weeks of gestation).

### Statistical analysis

#### Factors associated with placental malaria infection at delivery

Baseline continuous variables were presented as medians and their interquartile ranges (IQR) and categorical variables as numbers and their percentage. To assess factors associated with placental malaria, baseline category logit regression models were used. Since we were unable to obtain placental samples for over 40% of women recruited for the original study due to changes in delivery plans, we weighted observations to account for non-response bias by the inverse of the probability of women having placental samples (for both tests) as determined by a multivariable logistic regression model [27]. In building the multivariable models, variables were selected based on univariable analysis. Variables with *p*-values less than 0.2 were considered potential variables for the multivariable models (these included employment, gestational age at baseline, and BMI). Other covariates were included based on prior knowledge of the topic (years of education, wealth index, gravidity, mosquito prevention methods, anemia status). Due to the positive correlation between maternal age and parity in this population (ρ = 0.3 with *P* < 0.0001) maternal age was not included in any multivariable models. The trial regimens were forced into the multivariable models. Lastly, results were presented as odds ratio (OR) and their 95% confidence intervals.

#### The association between placental malaria and adverse pregnancy outcomes

In each outcome, the distributions of outcomes by exposure were presented in numbers and their percentage. To investigate the relationship of placental malaria with LBW, premature birth, and SGA birth, weighted log-binomial models, and log-Poisson models (with weights obtained from logistic regression models as described above) were used, [27, 28]. For HP placental malaria, the logistic regression model used to estimate the probabilities for weights was not stable due to a few cases in one of the age categories. In this case, we used the Firth penalized maximum likelihood estimation method [29]. All models were adjusted for employment status, years of education, wealth quartiles, trial regimen, gravidity, use of malaria interventions, twin pregnancy status, BMI, and anemia status at baseline. Results were presented using relative risk (RR) and their 95% confidence intervals.

Analyses were performed among women with both tests done. In this case, placental diagnoses were classified as HP placental malaria, sub-microscopic positive placental malaria when histopathology was negative, and negative for both diagnoses. The level of significance was 0.05, and all data analyses were conducted in SAS version 9.4 (SAS Institute Inc., Cary, NC, USA).

## Results

**Fig. 1:**
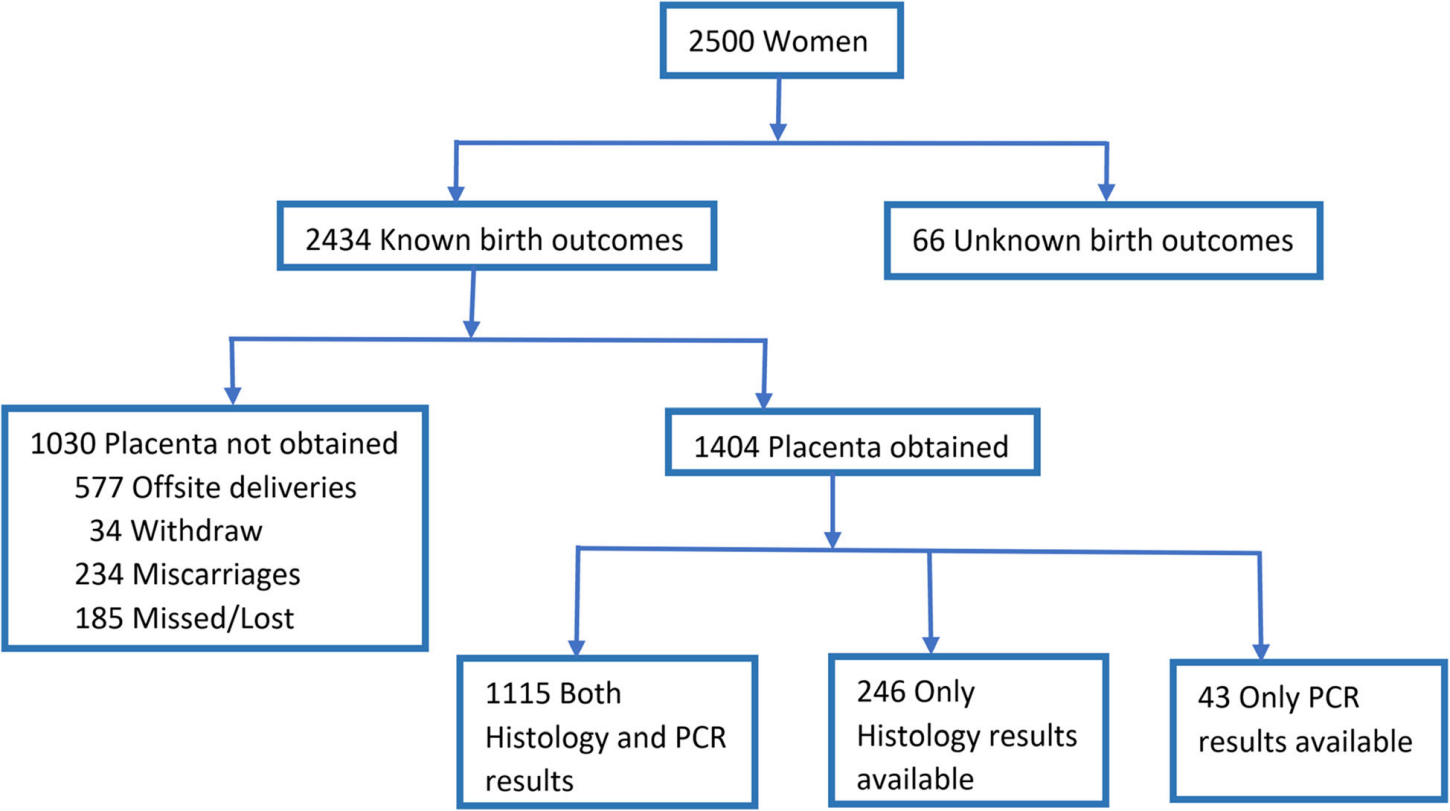
Participants flow chart

The original trial recruited 2500 pregnant women in their first trimester (Fig. 1). Of these 2434 had known birth outcomes and among those with known birth outcomes, we excluded 1030 women due to missing placentas. Among women with placenta samples, 1115 (79%) women had samples for both histology and PCR, 246 had samples only for histology and 43 had samples only for DNA PCR. Baseline characteristics in the groups of women with and without placenta samples were similar (Table 1). Among women included in the analysis, at baseline, the median age was 22 years with IQR [20–25] and the median gestational age was 10 weeks with IQR [8–12] (Table 2). Further, the majority were unemployed (53%), married or cohabiting (90%), with 0–7 years of education level (72%). Almost all women had singleton pregnancies (98%) and 93% used either fumigation or mosquito coils or bed nets at night. Underweight and peripheral malaria was less frequent (11 and 2% respectively), while anemia was very common (29%).

**Table 1:** The distribution of baseline characteristics among pregnant women with and without placenta sample for placental malaria diagnosis, 2010–2014 Dar-es-Salaam, Tanzania

Characteristics	Placenta sample	
	No (*n* = 1096)^a^	Yes (*n* = 1404)
Maternal age in years, median (IQR)	22 (20–25)	22 (20–25)
Maternal age in years, n (%)		
24 and below	815 (74.7)	993 (71.0)
25–34	268 (24.6)	388 (27.7)
35 and above	8 (0.7)	18 (1.3)
Gestational age in weeks^b^, median (IQR)	10 (8–12)	10 (8–12)
Employment, n (%)		
Employed^c^	326 (29.9)	415 (29.9)
Unemployed	620 (56.9)	764 (54.9)
Other	144 (13.2)	214 (15.4)
Marital status, n (%)		
Living single^d^	100 (9.2)	141 (10.1)
Married or cohabitating	989 (90.8)	1255 (89.9)
Years of education, n (%)		
0–7	774 (70.7)	1013 (72.2)
8–11	270 (24.7)	316 (22.5)
12 and above	51 (4.7)	75 (5.3)
Wealth quartile, n (%)		
1 (Lowest)	243 (23.7)	347 (26.4)
2	259 (25.3)	306 (23.3)
3	317 (30.9)	402 (30.6)
4 (Highest)	206 (20.1)	261 (19.8)
Number of previous pregnancies, n (%)		
First	530 (48.4)	668 (47.7)
Second	565 (51.6)	734 (52.4)
Twin pregnancy, n (%)		
Not twin	1080 (98.5)	1372 (97.7)
Twin	16 (1.5)	32 (2.3)
Uses fumigation or mosquito coils or net at night, n (%)		
No	64 (5.9)	87 (6.3)
Yes	1021 (94.1)	1304 (93.8)
Mid-upper arm circumference in cm, median (IQR)	26 (24–28)	26 (24–28)
Body mass index (kg/m^2^), median (IQR)	22.2 (20.2–25.4)	22.6 (20.1–25.7)
Body mass index (kg/m^2^), n (%)		
Underweight	112 (10.3)	151 (10.8)
Normal	681 (62.7)	843 (60.5)
Overweight or obese	293 (27.0)	400 (28.7)
Hemoglobin in g/dL, median (IQR)	11.6 (10.7–12.5)	11.6 (10.7–12.4)
Anemia status, n (%)		
Not anemic (Hb ≥ 11.0 g/dL)	738 (69.9)	938 (69.6)
Anemic (Hb < 11.0 g/dL)	318 (30.1)	410 (30.4)
Peripheral malaria, n (%)		
Negative	740 (98.0)	1305 (98.1)
Positive	15 (2.0)	26 (2.0)

^a^ Number of women excluded from the analysis due to missing placenta sample

^b^ Gestational age at enrollment based on the date of the last menstrual period

^c^ Employed includes skilled, unskilled, and informal employment

^d^ Living single includes never married, divorced, separated, and widow

Note: Percentage may be slightly below or above 100% because of rounding

**Table 2:** Distribution of baseline characteristics of pregnant women by placental malaria outcome, 2010–2014, Dar-es-Salaam, Tanzania

Characteristics	Placental test results			Total
	Negative^a^	DNA PCR Positive	Histopathology positive	
Overall, n (%)	886 (79.5)	136 (12.2)	93 (8.3)	1115 (100)
Maternal age in years, median (IQR)	22 (20–25)	22 (20–26)	22 (20–25)	22 (20–25)
Maternal age in years, n (%)				
≤ 24	624 (70.6)	91 (67.9)	65 (69.9)	780 (70.2)
25–34	249 (28.2)	40 (29.9)	27 (29.0)	316 (28.4)
≥ 35	11 (1.2)	3 (2.2)	1 (1.1)	15 (1.4)
Gestational age in weeks^b^, median (IQR)	10.6 (8.4–12.1)	10.1 (8.0–12.4)	11.3 (8.9–12.6)	10.4 (8.3–12.3)
Employment, n (%)				
Employed^c^	283 (32.3)	32 (23.7)	25 (27.2)	340 (30.8)
Unemployed	451 (51.4)	79 (58.5)	60 (65.2)	590 (53.4)
Other	143 (16.3)	24 (17.8)	7 (7.6)	174 (15.8)
Marital status, n (%)				
Living single^d^	92 (10.5)	11 (8.1)	9 (9.7)	112 (10.1)
Married or cohabitating	787 (89.5)	124 (91.9)	84 (90.3)	995 (89.9)
Years of education, n (%)				
0–7	630 (71.1)	106 (77.9)	71 (76.3)	807 (72.4)
8–11	198 (22.4)	25 (18.4)	21 (22.6)	244 (21.9)
≥ 12	58 (6.5)	5 (3.7)	1 (1.1)	64 (5.7)
Wealth quartile, n (%)				
1 (Lowest)	227 (27.6)	33 (25.8)	19 (21.8)	279 (26.9)
2	194 (23.5)	22 (17.2)	24 (27.6)	240 (23.1)
3	252 (30.6)	43 (33.6)	23 (26.4)	318 (30.6)
4 (Highest)	151 (18.3)	30 (23.4)	21 (24.1)	202 (19.4)
Number of previous pregnancies, n (%)				
First	430 (48.6)	63 (46.3)	44 (47.3)	537 (48.2)
Second	454 (51.4)	73 (53.7)	49 (52.7)	576 (51.8)
Twin pregnancy, n (%)				
Not twin	864 (97.5)	136 (100)	90 (96.8)	1090 (97.8)
Twin	22 (2.5)	0	3 (3.2)	25 (2.2)
Uses fumigation or mosquito coils or net at night, n (%)				
No	55 (6.3)	13 (9.8)	7 (7.6)	75 (6.8)
Yes	824 (93.7)	120 (90.2)	85 (92.4)	1029 (93.2)
Mid-upper arm circumference in cm, median (IQR)	26.0 (24.0–28.0)	26.0 (24.5–28.7)	27.0 (24.3–28.8)	26.0 (24.0–28.0)
Body mass index (kg/m^2^), median (IQR)	22.5 (20.1–25.9)	23.5 (20.9–25.8)	23.4 (20.9–26.7)	22.8 (20.3–25.9)
Body mass index (kg/m^2^), n (%)				
Underweight	106 (12.1)	5 (3.7)	7 (7.5)	118 (10.6)
Normal	515 (58.5)	84 (61.8)	55 (59.1)	654 (59.0)
Overweight or obese	259 (29.4)	47 (34.5)	31 (33.3)	337 (30.4)
Hemoglobin in g/dL, median (IQR)	11.7 (10.8–12.4)	11.4 (10.7–12.2)	11.5 (10.4–12.5)	11.6 (10.8–12.4)
Anemia status, n (%)				
Not anemic (Hb ≥ 11.0 g/dL)	612 (72.0)	88 (66.7)	58 (63.7)	758 (70.6)
Anemic (Hb < 11.0 g/dL)	238 (28.0)	44 (33.3)	33 (36.3)	315 (29.4)
Peripheral malaria, n (%)				
Negative	832 (98.5)	131 (100)	79 (90.8)	1042 (98.0)
Positive	13 (1.5)	0	8 (9.2)	21 (2.0)

^a^ Placental malaria test results negative for both Histopathology and PCR tests

^b^ Gestational age at enrollment based on the date of the last menstrual period

^c^ Employed includes skilled, unskilled, and informal employment

^d^ Living single includes never married, divorced, separated, and widow

Note: Percentage may be slightly below or above 100% because of rounding

Among 1404 women, there were 1115 (79%) pregnant women with sufficient placenta samples for both diagnostic methods, 886 (80%) tested negative by both methods, 93 (8%) had HP placental malaria, and 136 (12%) had sub-microscopic placental malaria.

### Factors associated with placental malaria infection at delivery

The distribution of baseline characteristics was presented in Table 2. According to both diagnostic methods, participants with placental malaria were more frequently unemployed (59% for DNA PCR and 65% for histopathology versus 51% for negative results), less educated (78% for DNA PCR and 76% for histopathology versus 71% for negative results), and anemic (33% for DNA PCR and 36% for histopathology versus 28% for negative results). Placental malaria was also somewhat more common among women not using mosquito prevention methods such as bed nets, fumigation, and coils (10% for DNA PCR and 8% for histopathology versus 6% for negative results).

Results of the multinomial model for factors associated with placental malaria are summarized in Table 3. Being employed and other forms of employment were associated with reduced odds of HP placental malaria compared to unemployed women (OR = 0.69; 95% CI: 0.47–1.00; *P* = 0.05 and OR = 0.49; 95% CI: 0.28–0.86; *P* = 0.01 respectively). Compared to those with a higher education level, pregnant women with the least education (0–7 years of education) had an increased odd of sub-microscopic placental malaria (OR = 2.21; 95% CI: 0.99–4.97; P = 0.05). Similar findings for HP placental malaria infection were observed but estimates were not stable due to the low number of outcomes. Pregnant women who did not use mosquito prevention methods such as fumigation, mosquito coil, or a bed net at night had a 75% increased risk of sub-microscopic placental malaria (OR = 1.75; 95% CI: 1.05–2.92; *P* = 0.03).

**Table 3:** Factors associated with placental malaria among women with sample for both diagnosis, 2010–2014, Dar es Salaam, Tanzania (*n* = 941)^a, b^

Characteristics	DNA PCR positive		Histopathology positive	
	OR [95% CI]	*P*-value	OR [95% CI]	*P*-value
Maternal age in years				
≤ 24	Ref		Ref	
≥ 25	1.20 [0.88, 1.64]	0.24	1.15 [0.79, 1.68]	0.46
Gestational age in weeks^c^	0.97 [0.92, 1.02]	0.23	1.08 [1.00, 1.15]	0.04
Employment				
Unemployed	Ref		Ref	
Employed^d^	0.72 [0.52, 1.00]	0.05	0.69 [0.47, 1.00]	0.05
Other	1.08 [0.75, 1.57]	0.67	0.49 [0.28, 0.86]	0.01
Years of education				
0–7	2.21 [0.99, 4.97]	0.05	5.87 [1.25, 27.51]	0.02
8–11	1.85 [0.80, 4.27]	0.15	6.48 [1.37, 30.68]	0.02
≥ 12	Ref		Ref	
Wealth index				
1 (Lowest)	Ref		Ref	
2	0.78 [0.52, 1.18]	0.24	1.58 [1.00, 2.50]	0.05
3	1.10 [0.77, 1.57]	0.62	0.91 [0.57 1.46]	0.69
4 (Highest)	1.22 [0.82, 1.82]	0.33	1.53 [0.94, 2.48]	0.09
Number of previous pregnancies				
First	0.94 [0.71, 1.25]	0.69	1.11 [0.79, 1.55]	0.56
Second	Ref		Ref	
Uses fumigation or mosquito coils or net at night				
No	1.75 [1.05, 2.92]	0.03	1.45 [0.76, 2.74]	0.26
Yes	Ref		Ref	
Body mass index (kg/m^2^)				
Underweight	0.33 [0.17, 0.62]	0.001	0.78 [0.44, 1.36]	0.38
Normal	Ref		Ref	
Overweight or obese	1.21 [0.90, 1.63]	0.21	1.48 [1.03, 2.11]	0.03
Anemia status				
Not anemic (Hb ≥ 11.0 g/dL)	Ref		Ref	
Anemic (Hb < 11.0 g/dL)	1.59 [1.20, 2.11]	0.001	1.70 [1.22, 2.38]	0.002

*OR* is an odds ratio

*CI* is the confidence interval

^a^ The model was adjusted for the trial supplement regimen received

^b^ The weighted analysis was used to account for missing placenta samples for placental malaria diagnosis. Multinomial distribution with the nominal outcome, each PCR positive and histopathology positive were compared to negative for both tests

^c^ Gestational age at enrollment based on last menstrual period

^d^ Employed includes skilled, unskilled, and informal employment

Being underweight was associated with reduced odds of sub-microscopic placental malaria compared to normal BMI (OR = 0.33; 95% CI: 0.17–0.62; *P* = 0.001). In contrast, being overweight/obese was associated with increased odds of HP placental malaria (OR = 1.48; 95% CI: 1.03–2.11; P = 0.03). Compared to pregnant women who were not anemic in the first trimester, anemic pregnant women had increased odds of sub-microscopic (OR = 1.59; 95% CI: 1.20–2.11; P = 0.001) and HP placental malaria (OR = 1.70; 95% CI: 1.22–2.38; *P* = 0.002).

### The association between placental malaria and adverse pregnancy outcomes

The prevalence of LBW was 8% among HP positive women and 5% among sub-microscopic positive women (Table 4). LBW was not associated with placental malaria by both diagnostic method. Premature births were common among women with placental malaria by both diagnostic methods (28 and 25% for HP and sub-microscopic placental malaria respectively). Compared to women negative for placental malaria, women with placental malaria infection had a slightly elevated risk of prematurity, although no statistical evidence for the association (RR = 1.16; CI: 0.89–1.52; *P* = 0.26 for HP placental malaria and RR = 1.14; CI: 0.90–1.45; *P* = 0.27 for sub-microscopic placental malaria).

**Table 4:** Relationship between low birth weight, premature, and small for gestational age births and placental malaria, 2010–2014, Dar es Salaam, Tanzania^a,b^

Exposure of interest	Proportion distribution of exposure n (%)		Univariable model		Multivariable model	
	Outcome	No outcome	RR [95% CI]	*P*-value	RR [95% CI]	*P*-value
**Low birth weight outcome**^**c**^						
Placental malaria			*n* = 922		n = 922	
Negative	72 (8.7)	756 (91.3)	Ref		Ref	
DNA PCR positive	7 (5.4)	123 (94.6)	0.46 [0.25, 0.85]	0.01	0.58 [0.23, 1.45]^d^	0.25
Histopathology positive	7 (7.8)	83 (92.2)	0.86 [0.50, 1.46]	0.57	0.90 [0.43, 1.90]^d^	0.79
**Premature births outcome**^e^						
Placental malaria			*n* = 917		n = 917	
Negative	175 (21.2)	651 (78.8)	Ref		Ref	
DNA PCR positive	32 (24.8)	97 (75.2)	1.10 [0.87, 1.40]	0.42	1.14 [0.90, 1.45]	0.27
Histopathology positive	24 (26.7)	66 (73.3)	1.18 [0.90, 1.53]	0.23	1.16 [0.89, 1.52]	0.26
**Small for gestational age outcome**^e^						
Placental malaria			*n* = 892		n = 892	
Negative	144 (18.0)	656 (82.0)	Ref		Ref	
DNA PCR positive	16 (12.4)	113 (87.6)	0.60 [0.42, 0.86]	0.01	0.61 [0.43, 0.88]	0.01
Histopathology positive	20 (22.5)	69 (77.5)	1.25 [0.94, 1.65]	0.13	1.30 [0.98, 1.72]	0.07

*RR* is a relative risk

*CI* is the confidence interval

The negative placental test was referring to negative to both PCR and histopathology tests

^a^ All multivariable models were adjusted for employment, years of education, wealth quartiles, supplements regimen, gravidity, use of combination malaria preventions, twin pregnant status, BMI, and anemia status at baseline

^b^ Weighted models were used to account for missing placenta samples for placental malaria diagnosis

^**c**^ Low birth weight is defined as live born with a birth weight below 2.5 kg

^d^ Multivariate Poisson distribution was applied to estimate relative risk because of the log-binomial model did not converge with desired covariates

^e^ Premature births were defined as live born delivered before 37 weeks of gestation

^e^ Small for gestational age birth was defined as live born infants with a birth weight below 10th percentile for sex and gestational age, based on INTERGROWTH-21st standards

A high prevalence of SGA births was found in women with HP placental malaria (23%), while women with sub-microscopic placental malaria had a low prevalence of SGA births (12%). Compared to women with negative results for both tests, HP placental malaria infection was associated with increased risk of SGA births (RR = 1.30; 95% CI: 0.98–1.72; *P* = 0.07), while sub-microscopic infection was associated with reduced risk of SGA births (RR = 0.61; 95% CI: 0.43–0.88; *P* = 0.01).

## Discussion

The overall prevalence of placental malaria was 8, and 12% for HP and sub-microscopic placental malaria respectively. Being underweight was associated with a reduced risk of sub-microscopic placental malaria while being overweight/obese was associated with a higher risk of HP placental malaria infection. Not using anti-malarial preventive measures and being anemic were associated with increased risk of sub-microscopic placental malaria. We noted an increased risk of SGA births in women with HP placental malaria and a decreased risk of SGA among women with sub-microscopic placental malaria. There was no statistically significant increased risk of premature births in women with either HP or sub-microscopic placental malaria.

Increased premature births among women infected with placental malaria have been observed in Uganda [30] and Papua New Guinea [31]. The Papua New Guinea [31] study further showed that chronic placental malaria was associated with an increased risk of prematurity. A potential mechanism for the association being the stimulation of early labor by the active immune triggered in malaria-infected placentas [32]. However, in the current study, we found a non-significant increased risk of premature births among women with placental malaria compared to women without the infection. The difference could be partly explained by differences in malaria transmission rates. In general urban areas receive fewer infectious bites per year compared to rural settings [33], and in 2009 the entomological inoculation rate in Dar-es-Salaam was 1.3 infectious bites per person per year [34].

The occurrence of IUGR in pregnant women infected with malaria is related to nutrient supply to the fetus. A study of the histopathology of infected placentas showed that the thickening of the cytotrophoblastic membranes may interfere with the nutrient supply to the fetus, resulting in growth retardation [32]. The association we observed between HP placental malaria and SGA was consistent with studies done in Uganda [30], and Gambia [35], although the latter did not account for potential confounders. A study conducted in the Democratic Republic of Congo showed that IUGR was highly associated with three or more cumulative infections, and there was a 2–8-fold increased risk of IUGR among women with evidence of under-nutrition [36]. However, we could not investigate a similar interaction effect between nutritional status and infection in the current study because few women were classified as underweight.

Our findings that sub-microscopic placental malaria was associated with a reduced risk of SGA birth was similar to results from a study done in Benin [37] despite differences in diagnostic methods and timing. The potential mechanism for this finding is unknown, but we speculate that it may involve the acquisition of antibodies in the placenta that inhibited the binding of *P. falciparum*-infected erythrocytes to the placental receptor chondroitin sulfate A (CSA) [38]. High levels of immunoglobulin G (IgG) against one of the placental isolates were previously found to be associated with increased birth weight among women who experienced at least one episode of malaria during pregnancy [39]. Further, Duffy and Fried [38] found that antibodies to placental parasites were related to decreased placental parasitemia and increased birth weight. Although our study participants included women in their first pregnancy who presumably lack immunity to CSA-binding parasite [40], the relationship may be plausible in the context of chronic or repeated infections in the same pregnancy. Findings from a study done in Kenya, that assessed pregnancy-specific *P. falciparum* immune responses among primiparous women showed low-to-medium pregnancy-associated malaria IgG concentration among women with acute or chronic MiP, which was an indication of the development of the immune response to infection [41]. On other hand, malaria infection during early pregnancy has been linked to SGA births [42]. Sub-microscopic placental malaria infection being interpreted to be a recent infection [16], we also suspect that the infections would have been those that occurred in the weeks preceding birth. Therefore, the association observed could have happened by chance.

Being underweight weakens antibody-mediated anti-malarial immunity [3]. This study, however, found that underweight was associated with reduced odds of sub-microscopic placental malaria. A possible explanation of this finding could be that pregnant women with macronutrient deficiencies (i.e. energy, fat, and protein) are at increased risk of micronutrient deficiency especially iron deficiency [3]. This was found in a study of Indonesian and Ghanaian pregnant women, in which the risk of anemia during early pregnancy was higher in women with lower levels of BMI during early pregnancy [43]. Being underweight may be associated with a reduced risk of placental malaria because of concurrent iron deficiency during pregnancy, which has been associated with reduced risk of malaria infection even during pregnancy [3, 44, 45]. Others have noted that pregnant women who are underweight during early pregnancy may already have been exposed to malaria infection and acquired pregnancy-specific immunity that protects them from further infection later in the same pregnancy [46].

We also noted the increased odds of HP placental malaria in women classified as overweight/obese. This finding was similar to that of a previous study done in Sweden [47], which found an increased risk of severe malaria among obese participants compared to normal weight participants, even though the study focused on the general adult population. Given the increased burden of overweight and obesity among women of reproductive age in urban Africa [48], our findings suggest that malaria control programs should also consider the nutrition status of the targeted population.

Being anemic during the first trimester was associated with an increased risk of sub-microscopic placental malaria, consistent with a study done in Malawi [15], but inconsistent with a study done in Cameroon [49]. Since anemia was detected at recruitment there is a possibility that low hemoglobin levels observed at recruitment resulted from prior malaria infections. Malaria infection detected in the placenta typically first occurred in the periphery many months earlier (possibly in the first trimester) resulting in anemia. Therefore, establishing a temporal pathway for the association observed in the current study is not possible given the nature of the study.

Intermittent preventive treatment in pregnancy (IPTp) with SP is among the strategies used for preventing adverse pregnancy outcomes associated with MiP in Tanzania [50], and it is available as standard of care for women attending antenatal care clinics. It has been shown that SP-IPTp can treat even sub-microscopic infection, but does not prevent new infections [14]. Hence, other malaria vector control strategies such as mosquito coils [51], fumigation [52], and treated bed nets [52, 53] are also used for malaria control. We found that not using these preventive methods was associated with an increased risk of sub-microscopic placental malaria. These findings support the protective role of alternative malaria control measures [52, 54]. However, others have suggested that mosquito coils have low mosquito repellency ability [51], and their effect on malaria prevention is not well established [55].

To our knowledge, our study is one of the first large studies to investigate the associations of placental malaria and adverse pregnancy outcomes among women in their first and second pregnancy in urban Tanzania. However, the study has some limitations first, placental malaria was diagnosed at delivery. Establishing the temporal relationship between malaria and baseline characteristics such as maternal anemia and underweight is therefore challenging, especially if malaria infection may have happened before enrolment. However, past infections diagnosed at delivery are informative with respect to the presence of MiP [6]. We also had very few women diagnosed with peripheral malaria at baseline (*n* = 41). DNA PCR diagnosis for placental malaria, though highly sensitive compared to histopathology, may detect both viable, and non-viable or gametocytes [6], which may complicate the interpretation of our results. However, sub-microscopic gametocytes have an important role in malaria transmission and pregnant women have been found to be reservoirs of sub-microscopic gametocytes [12]. It has been suggested that the majority of sub-microscopic malaria infection may reflect recent infection based on genotyping findings [16]. Therefore, learning about the relevance of this infection on clinical outcomes is crucial. Also, gestational age was estimated using the last menstrual period. This method is known to be vulnerable to misclassification of prematurity compared to ultrasound [56, 57]. However, in Tanzania ultrasound is not routinely available and where available it is not affordable for the majority. Lastly, approximately half of the study participants did not have a placental sample for placental malaria infection. To address potential selection bias due to missing data, we applied a weighted analysis.

In summary, social determinants such as low educational level may influence the understanding and interpretation of anti-malaria messages delivered at antenatal clinics. It is crucial that these programs tailor their messages and campaigns such that even those with low or no education are well informed. Further, even though over 90% reported the use of at least one malaria preventive measure, more effort should be directed to pregnant women who do not use recommended preventive measures. The integration of nutrition services that target both extremes of the malnutrition spectrum is warranted. Lastly, further research is also needed to understand sub-microscopic placental malaria and the underlying malaria immune response mechanisms that might be linked with the protective effect of SGA births among those infected.

## Data Availability

The datasets generated and/or analyzed are not publicly available because they contain sensitive information of participants but are available from the corresponding author on reasonable request.

## Abbreviations

HP: Histology-positive
PCR: Polymerase Chain Reaction
SGA: Small-for-gestational age
IQR: Interquartile range
RR: Relative risk
CI: Confidence interval
*P.falciparum*: *Plasmodium falciparum*
MiP: Malaria infection during pregnancy
HIV: Human immunodeficiency virus
SP: Sulphadoxine-pyrimethamine
LBW: Low birth weight
BMI: Body mass index
RNA: Ribonucleic acid
qRT-PCR: Quantitative real-time polymerase chain reaction
IUGR: Intrauterine growth retardation
IgG: Immunoglobulin G
CSA: Chondroitin sulfate A
IPTp: Intermittent preventive treatment in pregnancy
